# Geroderma Osteodysplasticum Due to a Recurrent Golgi Ras-Associated Binding (GORAB) GTPase-Binding Protein Variant: A Report of Two Cases From Morocco

**DOI:** 10.7759/cureus.111720

**Published:** 2026-06-29

**Authors:** Asmae Baaziz, Asmaa Mdaghri Alaoui

**Affiliations:** 1 Dysmorphology Unit, Department of Pediatrics P2, Children's Hospital, Ibn Sina University Hospital Center, Mohammed V University, Rabat, MAR

**Keywords:** consanguinity, cutaneous laxity, factor v deficiency, founder effect, geroderma osteodysplasticum, gorab, joint hyperlaxity

## Abstract

Geroderma osteodysplasticum (GO) is an ultra-rare inherited connective tissue disorder. We report a retrospective case series of two Moroccan patients with geroderma osteodysplasticum carrying the same recurrent Golgi Ras-associated binding (RAB) GTPase-binding protein (GORAB) variant. Patient 1 was an 11-year-old boy who presented in January 2026, and Patient 2 was a five-year-old girl who presented in September 2024; both were born to consanguineous parents. They harbored the identical homozygous pathogenic truncating GORAB variant (NM_152281.3:c.79C>T; p.Arg27*), confirmed by targeted Sanger sequencing and clinical exome sequencing, respectively, and classified as pathogenic in ClinVar (rs770355472). One of these patients (Patient 2) had previously been reported as an isolated case. Both patients presented with the classical triad of cutaneous laxity with pseudo-aged skin, generalized joint hyperlaxity, and growth retardation. Patient 1 additionally exhibited cryptorchidism and delayed bone age. Patient 2 presented with severe factor V deficiency (<1% activity), thoracolumbar kyphoscoliosis with vertebral wedging, and a bleeding diathesis characterized by recurrent epistaxis and easy bruising. Management was primarily supportive and multidisciplinary, including clinical monitoring and treatment tailored to individual manifestations. Follow-up demonstrated persistence of the underlying connective tissue disorder with variable clinical severity but no major disease-specific complications. The identification of the identical GORAB p.Arg27* variant in two unrelated Moroccan consanguineous families raises the possibility of a founder effect warranting further population-based investigation. By comparing two phenotypically distinct patients carrying the same pathogenic variant, this report highlights the clinical variability associated with GORAB-related disease and suggests that hemostatic evaluation should be considered in patients with GO who present with a personal or family history suggestive of a bleeding disorder.

## Introduction

Geroderma osteodysplasticum (GO; OMIM #231070), also known as Walt Disney dwarfism, is an exceptionally rare autosomal recessive connective tissue disorder. Its molecular basis was subsequently characterized by Hennies et al. [1]. The condition is caused by biallelic loss-of-function variants in the Golgi Ras-associated binding (RAB) GTPase-binding protein (GORAB) gene (Golgin, RAB6-Interacting; OMIM #607983), located on chromosome 1q24.2, which encodes a Golgi-associated protein involved in vesicular trafficking and extracellular matrix (ECM) protein secretion, particularly of collagen and elastin [1,2].

The clinical phenotype of GO is distinctive, encompassing generalized cutaneous laxity with a prematurely aged and wrinkled appearance of the skin, joint hyperlaxity affecting both large and small joints, significant growth retardation, osteoporosis with a predisposition to vertebral fractures and compression, and mild facial dysmorphism. The combination of these features, particularly the aged skin appearance in a child, is highly suggestive of the diagnosis when interpreted in the appropriate clinical, familial, and genetic context [1,3]. Clinical manifestations are typically evident from infancy or early childhood, with cutaneous laxity and pseudo-aged skin frequently recognized within the first year of life. However, the rarity of the condition and its phenotypic overlap with other connective tissue disorders, including Ehlers-Danlos syndrome and cutis laxa, may result in substantial diagnostic delays, as illustrated by the relatively late diagnosis of Patient 1 at 11 years of age in the present series. Early recognition of GO is important to facilitate appropriate multidisciplinary management, genetic counseling, and surveillance for disease-related complications.

To date, fewer than 60 cases of GO have been reported in the global literature, with a significant proportion originating from consanguineous families in the Middle East, Turkey, and Pakistan [3,4]. The GORAB p.Arg27* truncating variant (c.79C>T), caused by a nonsense mutation in exon 2, has been identified as a recurrent allele in several unrelated families, suggesting the existence of a potential founder effect in specific geographic or ethnic populations [5,6]. To date, only one molecularly confirmed case from Morocco has been reported, describing the coexistence of GO and severe factor V deficiency [7].

We present two unrelated Moroccan children from consanguineous families, both harboring the identical homozygous GORAB p.Arg27* variant, with overlapping but phenotypically distinct presentations. Patient 2 was previously reported as a single case [7] and is included in the present series to enable comparison with a second unrelated Moroccan patient carrying the same pathogenic variant, with the aim of documenting phenotypic variability and exploring the possibility of a founder effect for the GORAB p.Arg27* allele in the Moroccan population.

## Case presentation

This retrospective case series included two consecutive patients diagnosed with geroderma osteodysplasticum who presented to the Dysmorphology Unit, Department of Pediatrics P2, Children's Hospital, Ibn Sina University Hospital, Rabat, Morocco, between September 2024 and January 2026. Both patients were included based on molecular confirmation of the diagnosis.

Patient 1: clinical presentation

Patient 1 is an 11-year-old Moroccan boy, born to second-cousin consanguineous parents, referred to the dysmorphology and genetics consultation for suspected genodermatosis with osteodysplastic features. The pregnancy was carried to term without reported complications. Psychomotor development was reported as normal. Both parents were phenotypically unaffected and did not exhibit cutaneous, skeletal, or articular manifestations consistent with GO. Family history was negative for similarly affected relatives, and no other family members were reported to have connective tissue abnormalities, growth retardation, or comparable dermatological manifestations. Parental genetic testing was not available; therefore, segregation analysis could not be performed.

Anthropometric measurements revealed a weight of 29 kg (approximately the 15th percentile) and a height of 131 cm (third percentile for age and sex according to the WHO Growth Reference for School-aged Children and Adolescents), indicating significant short stature. Head circumference measured 52 cm.

**Figure 1:**
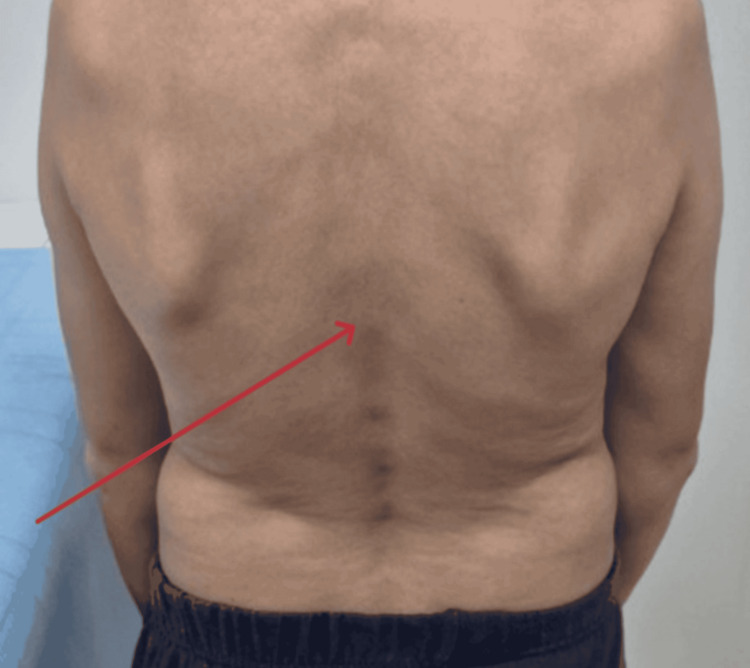
Posterior view of Patient 1 showing prominent rib reliefs and scapulae alatae (arrow), consistent with the skeletal manifestations of geroderma osteodysplasticum.

**Figure 2:**
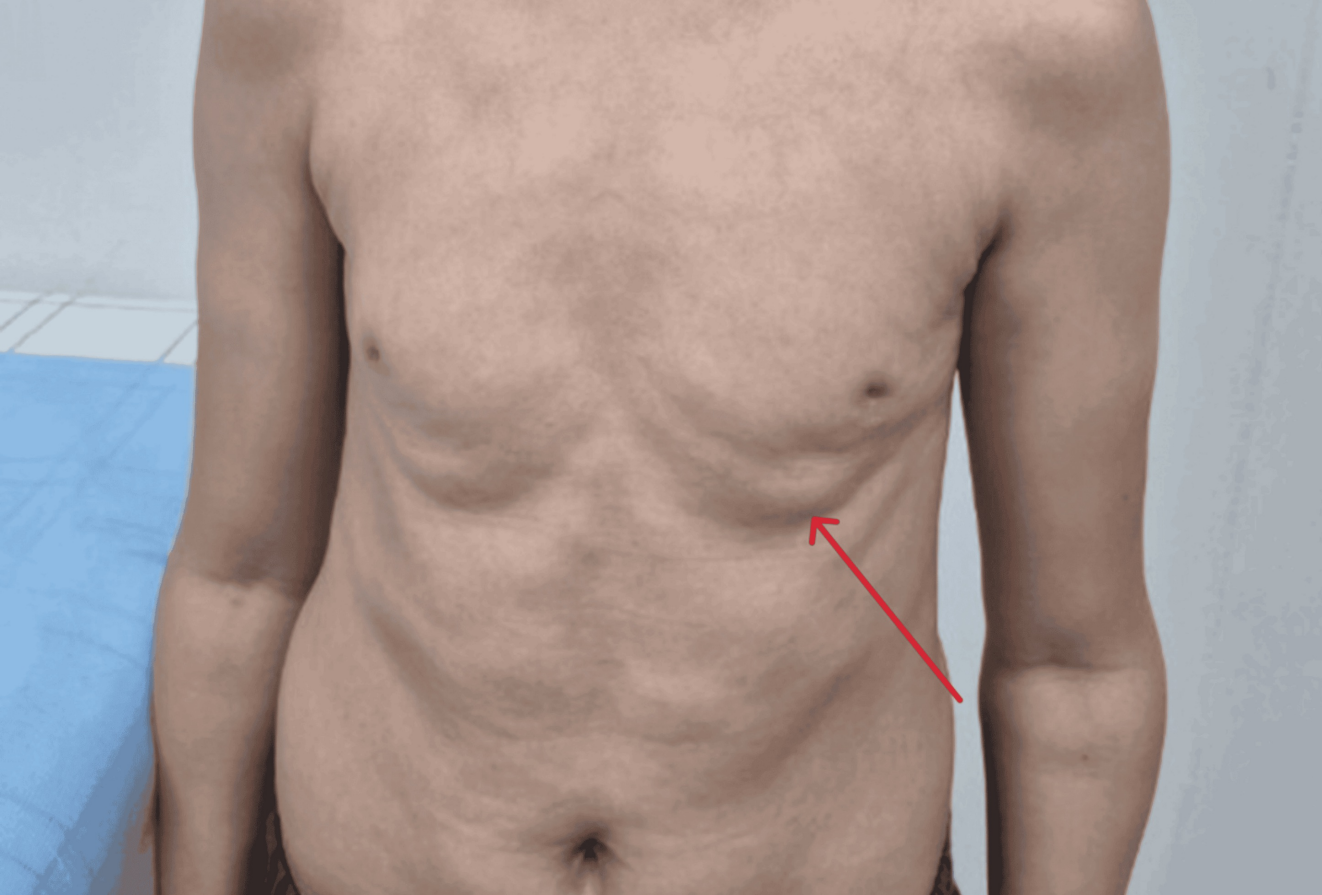
Frontal view of Patient 1 showing near-total absence of subcutaneous adipose tissue giving a lipodystrophic appearance, with prominent rib reliefs (arrow), consistent with geroderma osteodysplasticum.

Dysmorphological examination demonstrated a mildly dysmorphic facies characterized by low-set, posteriorly rotated, and prominent ears, mildly deep-set eyes, and a generally aged facial appearance. Cutaneous examination revealed markedly thin, wrinkled, and finely folded skin with an overall pseudo-aged appearance and near-total absence of subcutaneous adipose tissue, imparting a striking lipodystrophic phenotype. Musculoskeletal assessment demonstrated generalized joint hyperlaxity. Thoracic examination revealed a mild chest wall deformity with prominent ribs and scapular winging (scapulae alatae) (Figures 1, 2).

Additional findings included right testicular cryptorchidism, confirmed on inguinoscrotal ultrasonography. Skeletal maturation, assessed by left-hand radiography using the Greulich and Pyle method performed when the patient was nine years of age (two years prior to the current evaluation), revealed a bone age of approximately eight years, indicating a delay of one year relative to chronological age at the time of assessment. Brain computed tomography (CT) was unremarkable. Abdominal ultrasonography was normal. Thyroid function tests were within normal limits, with a thyroid-stimulating hormone (TSH) of 3 mIU/L (reference range: 0.5-4.5 mIU/L) and a free T4 of 15 pmol/L (reference range: 10-23 pmol/L). Whole-body skeletal evaluation was performed with a skeletal survey consisting of standard radiographs, which revealed no additional significant osseous abnormalities beyond the findings described above.

Patient 1: genetic analysis

Targeted Sanger sequencing of exon 2 of the GORAB gene (RefSeq: NM_152281.3) was performed at a certified molecular genetics laboratory using an ABI 3500 Genetic Analyzer (Thermo Fisher Scientific, Waltham, MA, USA). The sequencing covered the coding sequence of exon 2, including flanking intron-exon boundaries. Analysis identified the homozygous recurrent truncating variant NM_152281.3(GORAB):c.79C>T (p.Arg27*). This variant is classified as pathogenic according to the American College of Medical Genetics and Genomics (ACMG) guidelines and confirmed the diagnosis of GO (Table 1).

**Table 1:** Comparative clinical and genetic features of the two patients. GORAB: Golgi Ras-associated binding.

Feature	Patient 1	Patient 2
Sex	Male	Female
Age at diagnosis	11 years	Five years
Consanguinity	Second-cousin consanguinity	First-cousin consanguinity
Growth retardation	Yes	Yes
Cutaneous laxity/pseudo-aged skin	Yes-marked	Yes
Lipodystrophy	Yes-marked	Yes
Joint hyperlaxity	Diffuse	Peripheral
Delayed bone age	~1 year	-
Cryptorchidism	Yes-right	N/A
Factor V deficiency	Not tested	Severe (<1%)
Bleeding history	Not reported	Epistaxis, easy bruising
GORAB variant	c.79C>T p.Arg27* homozygous	c.79C>T p.Arg27* homozygous
Genetic method	Targeted Sanger sequencing	Clinical exome sequencing
ClinVar classification	Pathogenic	Pathogenic

Management and follow-up

Following molecular confirmation of the diagnosis, Patient 1 was enrolled in a multidisciplinary follow-up program. Management included vitamin D supplementation and bisphosphonate therapy to improve bone mineralization and reduce the risk of fractures, along with regular rheumatologic assessment and physiotherapy. At the latest follow-up visit, the patient remained clinically stable, with no new fractures or significant disease progression reported.

Patient 2: clinical presentation

**Figure 3:**
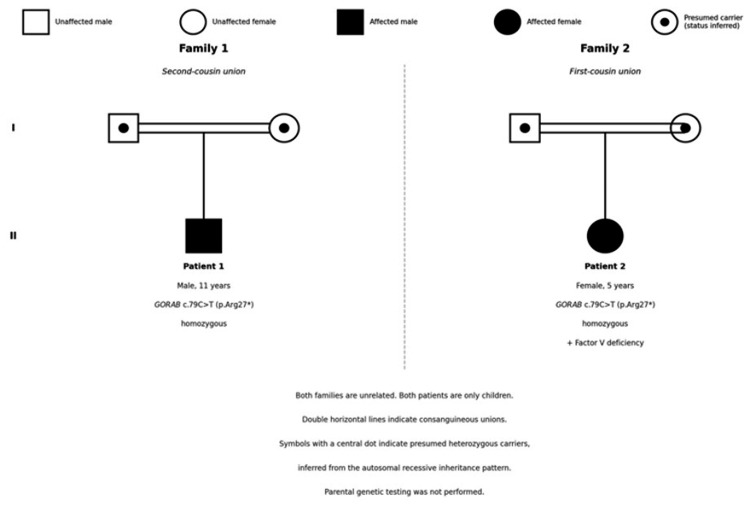
Pedigrees of two unrelated consanguineous Moroccan families with geroderma osteodysplasticum. Pedigrees of Family 1 and Family 2. Family 1 shows a second-cousin union, and Family 2 shows a first-cousin union, both indicated by double horizontal lines. Filled symbols represent affected individuals: Patient 1 (Family 1), an 11-year-old boy harboring the homozygous GORAB c.79C>T (p.Arg27*) variant; and Patient 2 (Family 2), a five-year-old girl carrying the same homozygous variant with an additional diagnosis of factor V deficiency. Symbols with a central dot denote presumed obligate heterozygous carriers, inferred from the autosomal recessive inheritance pattern. Both patients are only children. Parental genetic testing was not performed. The two families are unrelated. Figure is a pedigree diagram generated programmatically using Python (version 3.x) (Python Software Foundation, Wilmington, DE, USA) with the matplotlib library. It was created using a custom script to accurately represent the family structure of the reported cases.

Patient 2 is a five-year-old Moroccan girl, born to first-cousin consanguineous parents, referred to the pediatric genetics consultation with an initial clinical suspicion of Ehlers-Danlos syndrome based on prominent joint hyperlaxity. She was previously reported by our group in a preliminary case report [7]. She is included in the present series for two reasons. First, her clinical presentation differs substantially from that of Patient 1, notably through the coexistence of severe factor V deficiency, a finding not previously reported in association with GO and therefore of particular clinical interest. Second, the identification of the same homozygous GORAB c.79C>T (p.Arg27*) variant in two unrelated consanguineous Moroccan families raises the possibility of a founder effect for this recurrent variant in the Moroccan population. Growth retardation had been noted since early childhood. Both parents were phenotypically unaffected and did not exhibit cutaneous, skeletal, or articular manifestations consistent with GO. Family history was negative for similarly affected relatives, and no other family members were reported to have connective tissue abnormalities, growth retardation, or comparable dermatological manifestations. Parental genetic testing was not available; therefore, segregation analysis could not be performed. The pedigrees of both consanguineous families are shown in Figure 3.

**Figure 4:**
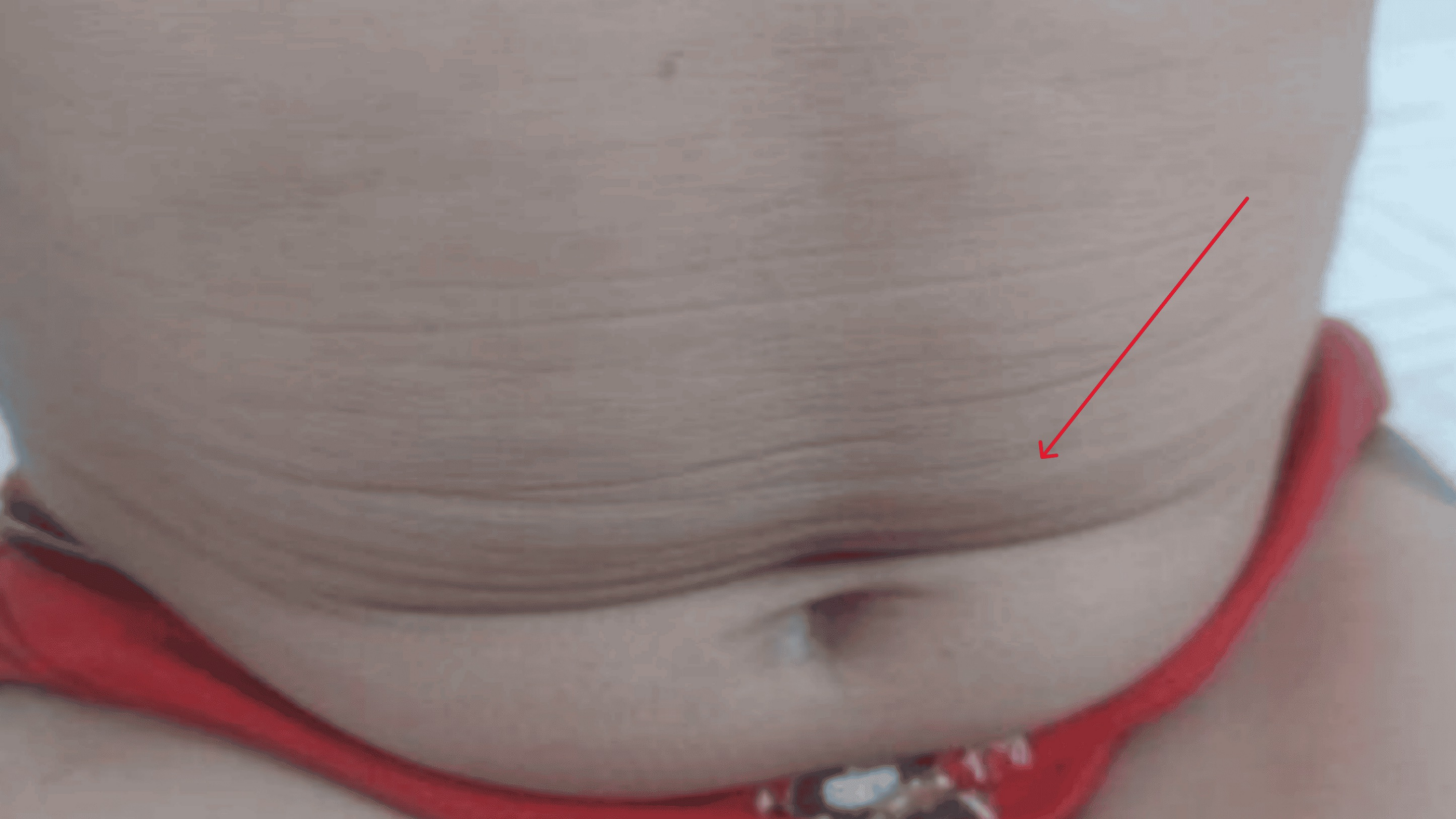
Marked generalized skin laxity with prominent abdominal folds at rest in Patient 2, a hallmark feature of geroderma osteodysplasticum (arrow).

Clinical examination demonstrated a mildly dysmorphic facies with large eyes, thin facial features, and sparse hair with a low-set anterior hairline. Cutaneous examination revealed marked generalized skin laxity with prominent abdominal folds at rest, increased skin extensibility on pinching, and fine cutaneous wrinkling with near-total absence of subcutaneous adipose tissue, imparting a lipodystrophic appearance, most prominent over the abdomen and scalp (Figure 4).

Marked peripheral joint hyperlaxity was present, particularly at the ankles and feet. Thoracic and spinal examination revealed exaggerated thoracic kyphosis and mild thoracolumbar scoliosis with vertebral wedging; original imaging files were unavailable for inclusion, and findings, including vertebral wedging, are reported based on written radiological reports.

Hemostatic evaluation revealed a severely reduced factor V activity of <1% of normal (reference range: 62-139%), in the context of recurrent epistaxis and easy bruising. Coagulation studies showed a markedly decreased prothrombin time (PT: 25.1%; international normalized ratio (INR): 3.57) and a prolonged activated partial thromboplastin time (aPTT: 91.1 s; reference range: 23-38 s), consistent with severe factor V deficiency. Factor VII activity was 71% (reference range: 50-129%), factor VIII activity was 95% (reference range: 50-150%), and factor X activity was 84% (reference range: 77-131%), all within normal limits. Fibrinogen concentration was 2.99 g/L (reference range: 1.89-4.75 g/L). The normal factor VIII activity excludes combined factor V and factor VIII deficiency. This coagulation profile is consistent with severe isolated inherited factor V deficiency, a finding not previously reported in a patient with GO. Although the coexistence of these two rare conditions is intriguing, the present observation does not establish a causal relationship and requires confirmation in additional patients.

Patient 2: genetic analysis

Clinical exome sequencing (CES_v3; Sophia Genetics, Saint-Sulpice, Switzerland) was performed at a certified molecular genetics laboratory using an Illumina NovaSeq 6000/NextSeq 500/550 platform (Illumina, Inc., San Diego, CA, USA), covering the coding regions (±5 bp of intronic boundaries) of 5,500 genes and the mitochondrial genome. Variant interpretation was performed using Sophia DDM® V5.10.34 (Sophia Genetics, Saint-Sulpice, Switzerland). A phenotype-directed in silico filter covering 21 genes associated with Ehlers-Danlos syndrome was initially applied, and no pathogenic variants were identified within this targeted panel. Expanded exome analysis subsequently identified the homozygous pathogenic truncating variant NM_152281.3(GORAB):c.79C>T p.(Arg27*), with a sequencing read depth of 54×, classified as pathogenic in ClinVar (rs770355472) per ACMG criteria, thereby confirming the diagnosis of GO.

Management and follow-up

Following molecular confirmation of the diagnosis, Patient 2 was enrolled in a multidisciplinary follow-up program. Management included vitamin D supplementation and bisphosphonate therapy to optimize bone health. Given the coexisting severe factor V deficiency, regular hematological follow-up was instituted, and fresh frozen plasma was recommended prophylactically before any invasive procedure. No major hemorrhagic episodes have been reported during follow-up. At the most recent evaluation, the patient remained clinically stable without significant disease-related complications.

The comparative clinical and genetic features of both patients are summarized in Table 1. Laboratory investigations for both patients are summarized in Table 2.

**Table 2:** Laboratory investigations of Patient 1 and Patient 2. PT: prothrombin time; INR: international normalized ratio; aPTT: activated partial thromboplastin time; TSH: thyroid-stimulating hormone; free T4: free thyroxine; ND: not done.

Test	Patient 1	Patient 2	Reference range
TSH (mIU/L)	3.0	ND	0.5-4.5
Free T4 (pmol/L)	15	ND	10-23
PT (%)	ND	25.1	>70
INR	ND	3.57	0.8-1.25
aPTT (s)	ND	91.1	23-38
Factor V activity (%)	ND	<1	62-139
Factor VII activity (%)	ND	71	50-129
Factor VIII activity (%)	ND	95	50-150
Factor X activity (%)	ND	84	77-131
Fibrinogen (g/L)	ND	2.99	1.89-4.75

## Discussion

We report two unrelated Moroccan children with molecularly confirmed geroderma osteodysplasticum (GO), both carrying the identical homozygous GORAB c.79C>T (p.Arg27*) truncating variant. Patient 2 was previously reported as an isolated case [7]; the present series constitutes the first multi-case report of GO from Morocco and the Maghreb region, and the first description of the GORAB p.Arg27* allele in two unrelated North African families.

The GORAB gene encodes a Golgi-associated protein that interacts with RAB6 and plays a critical role in vesicular trafficking and the secretion of extracellular matrix proteins, particularly collagen and elastin [1,3]. Loss-of-function variants such as p.Arg27* are predicted to result in nonsense-mediated mRNA decay and complete loss of protein function, leading to impaired extracellular matrix assembly and maintenance. This molecular mechanism explains the characteristic cutaneous laxity, skeletal fragility, growth retardation, and connective tissue abnormalities observed in affected individuals [3].

The recurrent identification of the GORAB p.Arg27* variant in Turkish, Pakistani, Middle Eastern, and now Moroccan families suggests either a mutational hotspot or the presence of founder effects within specific populations [5,6]. The detection of the identical homozygous variant in two apparently unrelated Moroccan patients from consanguineous families strengthens the hypothesis of a regional founder effect. Although haplotype analysis would be required for formal confirmation, the present findings provide suggestive evidence supporting further population-based genetic investigations in Morocco and the broader North African region.

The clinical manifestations observed in both patients are highly consistent with the established phenotypic spectrum of GO. Both presented with the classical triad of cutaneous laxity with pseudo-aged skin, generalized joint hyperlaxity, and growth retardation. The striking aged appearance of the skin remains one of the most recognizable hallmarks of the disorder and reflects the underlying extracellular matrix defect. Skeletal involvement was prominent in both cases, including thoracic deformity in Patient 1 and thoracolumbar kyphoscoliosis with vertebral wedging in Patient 2, findings that are in keeping with the osteoporotic and dysplastic skeletal phenotype previously described in GO [4].

The diagnostic delay observed in Patient 1, who received molecular confirmation only at 11 years of age despite long-standing characteristic manifestations, highlights the challenges associated with recognizing ultra-rare connective tissue disorders. Although the clinical features of GO are typically evident from infancy or early childhood, diagnosis may be delayed because of limited disease awareness, phenotypic overlap with other hereditary connective tissue disorders, and restricted access to molecular genetic testing. Early recognition of the characteristic clinical triad should prompt consideration of GO and facilitate timely molecular confirmation. Establishing the diagnosis at an early stage may improve multidisciplinary follow-up, genetic counseling, and surveillance for progressive skeletal complications.

The most notable finding in this series is the identification of severe factor V deficiency (<1% activity) in Patient 2. Factor V is a critical coagulation cofactor involved in thrombin generation through the prothrombinase complex [8]. Inherited factor V deficiency is a rare autosomal recessive bleeding disorder caused by pathogenic variants in the F5 gene and has an estimated prevalence of approximately one per million individuals [9]. The profound reduction in factor V activity observed in our patient adequately explains the history of recurrent epistaxis and easy bruising. To our knowledge, the coexistence of GO and severe inherited factor V deficiency has been reported only once previously, in the preliminary case report describing Patient 2 [7].

Given the high degree of consanguinity in the family, the most parsimonious explanation is the coexistence of two independent autosomal recessive conditions in the same individual. Such occurrences are increasingly recognized in highly consanguineous populations, where homozygosity for multiple pathogenic variants may occur simultaneously [6]. Molecular analysis of the F5 gene would be required to confirm this hypothesis and identify the underlying causal variant. Although the coexistence of GO and severe factor V deficiency is intriguing, the present report cannot establish a causal relationship between the two conditions. Additional cases and molecular studies will be necessary to determine whether this observation represents a true disease association or a coincidental occurrence.

The normal cardiac evaluation observed in Patient 2 is consistent with previous reports indicating that structural cardiac anomalies are not a recognized feature of GO. Nevertheless, echocardiographic assessment remains useful in the differential diagnostic workup, particularly to exclude cardiovascular manifestations associated with other connective tissue disorders such as Marfan syndrome and certain forms of Ehlers-Danlos syndrome [10]. The principal differential diagnoses of GO include cutis laxa syndromes, Ehlers-Danlos syndrome, De Barsy syndrome, and wrinkly skin syndrome. In both patients, identification of the pathogenic GORAB variant definitively established the diagnosis and resolved the diagnostic uncertainty.

The main limitations of this report include the small number of patients and the absence of haplotype analysis and F5 gene sequencing, which precluded confirmation of a founder effect and clarification of the genetic basis of the severe factor V deficiency observed in Patient 2.

## Conclusions

These two molecularly confirmed Moroccan cases of geroderma osteodysplasticum carrying the same GORAB p.Arg27* variant illustrate the clinical variability of the disorder despite an identical genotype. The association of severe factor V deficiency in one patient highlights the importance of considering hemostatic evaluation in patients with GO who present with bleeding manifestations. These cases also emphasize the value of molecular genetic testing for accurate diagnosis and multidisciplinary management in consanguineous families with connective tissue disorders.
